# Molecular identity of the mechanotransduction machinery in inner ear hair cells and mechanotransduction-linked hearing loss

**DOI:** 10.1016/j.fmre.2025.01.019

**Published:** 2025-02-28

**Authors:** Yanyan Jia, Huawei Li, Wenyan Li

**Affiliations:** a ENT Institute and Otorhinolaryngology Department of Eye & ENT Hospital, State Key Laboratory of Medical Neurobiology and MOE Frontiers Center for Brain Science, Fudan University, Shanghai 200031, China; b Institutes of Biomedical Sciences, Fudan University, Shanghai 200032, China; c NHC Key Laboratory of Hearing Medicine, Fudan University, Shanghai 200031, China; d The Institutes of Brain Science and the Collaborative Innovation Center for Brain Science, Fudan University, Shanghai 200032, China

**Keywords:** Auditory hair cell, Mechano-electrical transduction (MET), MET channel complex, TMC1 channel, Hearing loss

## Abstract

Hearing is among the most sensitive sensory modalities in vertebrates, and its dysfunction can lead to hearing loss. Auditory perception within the inner ear relies on mechanosensitive hair cells and a sophisticated network of molecules that comprise the machinery responsible for mechanotransduction. The mechanosensory hair cells exhibit distinct structural features that enable them to transform sound vibrations into electrical signals, which are then relayed to the spiral ganglion neurons (SGNs) that are connecting between the cochlear hair cells and the cochlear nuclei with high accuracy and fidelity. Despite over 40 years of research, the precise molecular composition and assembly of the mechanotransduction channel complex within cochlear hair cells remains elusive. This review presents recent findings that provide new perspectives on the molecular components and assembly of the mechanotransduction apparatus in cochlear hair cells, as well as genetic mutations related to mechanotransduction dysfunction and hearing loss.

## 1 Introduction

In mammals, the auditory process relies on cochlear hair cells that transform sound-induced mechanical forces into electrochemical signals. This transformation, referred to as mechano-electrical transduction (MET), begins when the hair bundle located at the top of the hair cells is deflected, opening the sensory transduction channels rapidly. The inward flow of potassium ions via these channels causes the depolarization of hair cells. This change in electrical charge initiates the release of neurotransmitters onto afferent nerve fibers, thereby transmitting auditory signals to the cochlear nuclei (CN) [1].

More than four decades ago, electrophysiological studies on vertebrate hair cells first uncovered the presence of mechanotransduction ion channels [2,3]. Since their discovery, scientists have diligently sought to identify the molecular components of these channels, essential for mechanically activated currents, although progress has been gradual. Since then, significant efforts have been made to identify the molecular components responsible for these channels, which are activated by mechanical stimuli. Despite the slow progress, genetic research over the last 30 years has identified several key proteins involved in the MET process in both mice and humans. These proteins include TMC1/2, LHFPL5, TMIE, CIB2/3, CDH23 and PCDH15, all of which are crucial for the proper functioning of hair cell MET machinery. Among these, TMC1/2 have been considered as the pore-forming subunits of the transduction channel, marking a significant advancement in understanding MET mechanisms. Despite these breakthroughs, critical questions remain, such as elucidating the specific roles of each component and how mechanical forces open these channels. It is anticipated that a more comprehensive insight into MET, spanning both biophysical and molecular scales, will be achieved within the next decade.

In this review, we provide an overview of the latest progress in auditory mechanotransduction. We will highlight the role of the key proteins within the MET machinery and explore additional components integral to the MET process, and discuss the assembly and gating mechanism of MET channel. Additionally, we will summarize genetic mutations related to MET dysfunction and hearing loss.

## 2 Cochlear hair cell and mechanotransduction

**Fig. 1:**
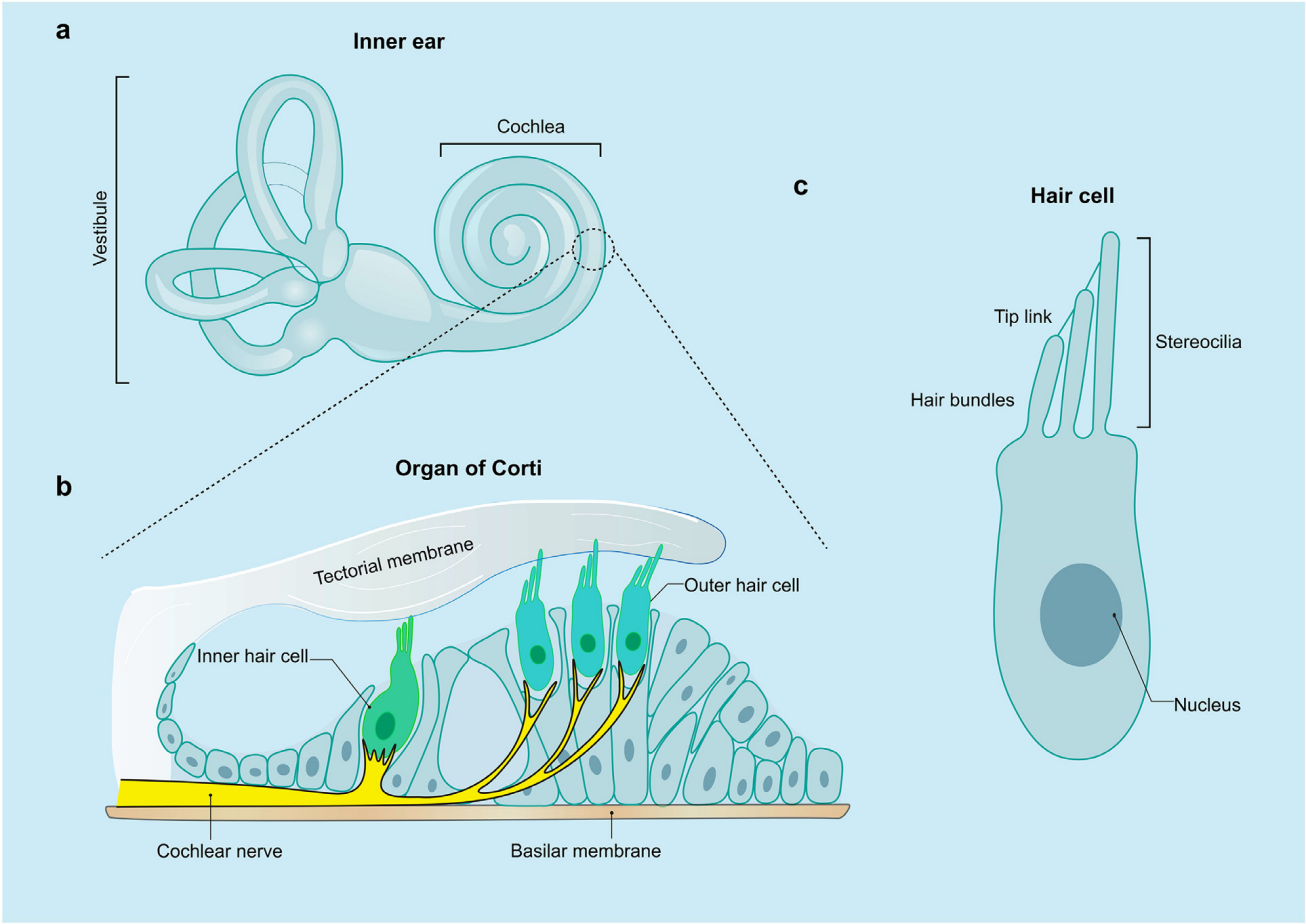
**Structure of the inner ear and organization of hair cells in the cochlea.** (a) The diagram highlights the inner ear structure, with the spiral-shaped cochlea responsible for converting sound vibrations into neural signals. (b) A cross-sectional view of the cochlear structure, focusing on the organ of Corti located on the basilar membrane. The organ of Corti contains both inner and outer hair cells. Inner hair cells (IHCs; one row) are responsible for transmitting auditory information to the brain through the cochlear nerve, while outer hair cells (OHCs; three rows) amplify sound. The tectorial membrane is positioned above the hair cells and interacts with the stereocilia during sound vibrations. The absence of spiral ganglion neurons (SGNs) and inner ear efferents (IEEs) leads to the loss of both IHCs and OHCs [128], while the elimination of IEEs primarily results in the deterioration of OHCs [129]. (c) A close-up of a single hair cell, showing its stereocilia (hair bundles) and tip links that connect adjacent stereocilia. The hair bundles, consisting of actin-filled stereocilia arranged in a staircase formation, are located on the apical surface of hair cells. Tip links are crucial for opening MET channels during stereocilia deflection, enabling ion flow, which results in hair cell depolarization and the conversion of sound into neural signals.

**Fig. 2:**
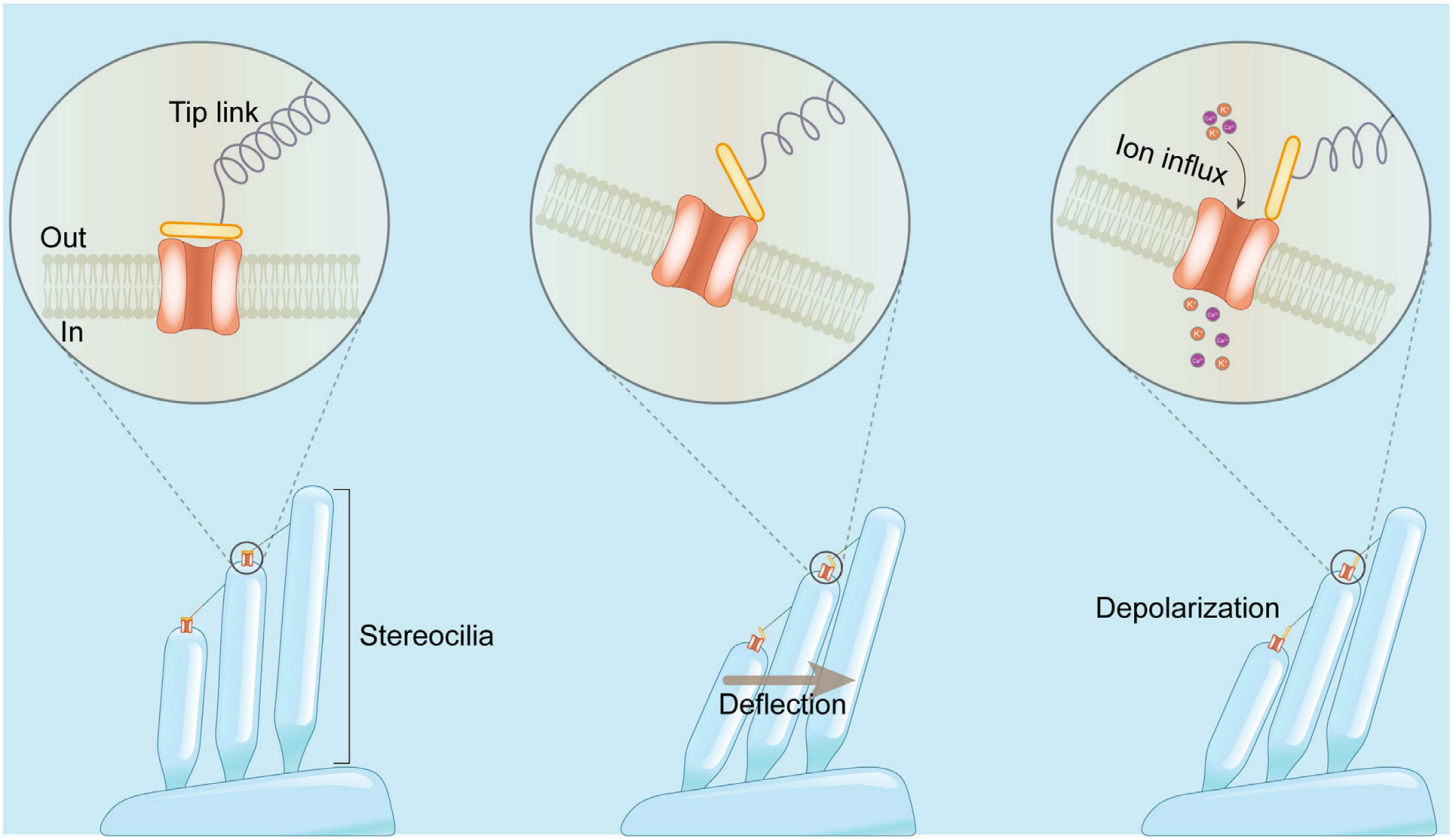
**Mechanotransduction in auditory hair cells.** This schematic illustrates the sequence of events in the mechanotransduction process in auditory hair cells. In response to sound, the stereocilia bundle deflects, exerting tension on the tip links connecting adjacent stereocilia (center panel). This mechanical force opens MET channels located at the tips of stereocilia, allowing the influx of K⁺ and Ca²⁺ ions (right panel). The ion influx depolarizes the cell membrane, initiating the signal transduction pathway that leads to neurotransmitter release and auditory signaling to the brain. Insets show detailed views of the MET channel, tip link, and direction of ion flow during the transduction process.

The auditory sensory epithelium in mammals resides within the organ of Corti, nestled in the cochlear spiral of the inner ear (Fig. 1). Within the cochlea, mammalian hair cells are categorized into two functional groups: inner hair cells (IHCs) and outer hair cells (OHCs) [1]. IHCs are responsible for transmitting auditory information to the brain by forming connections with afferent SGNs, while OHCs are essential for amplifying and refining incoming sound waves. Despite their differing functions, both types of hair cells share a common ability to convert mechanical stimuli to electrical signals, a key process in hearing referred to as mechano-electrical transduction (MET). The hair bundles, situated on the apical surface of each hair cell, are the specialized structures that responsible for this mechanotransduction. These hair bundles consist of rows of actin-rich protrusions referred to as stereocilia, organized in a characteristic staircase mode, with the shortest stereocilia at one end and progressively taller ones at the other. During early development, hair cells are also characterized by the presence of a kinocilium, which is filled with microtubules. In mature cochlear hair cells, however, this structure is no longer present [4]. The process of mechanotransduction begins as the movement of sound-induced waves along the basilar membrane cause the deflection of hair bundles. This deflection creates strain in the tip links, the fine filaments that link the top of one stereocilium to the side of its taller adjacent counterpart. The increased tension in the tip links initiates the MET channels located near the stereocilia tips to open, allowing the influx of K^+^ and Ca^2+^, into the hair cell. The ion influx causes the hair cell to become depolarized, prompting the activation and release of neurotransmitters at its base. This neurotransmitter release activates the SGNs, ultimately transmitting sound information to the cochlear nuclei (Fig. 2).

## 3 Architecture of cochlear hair cell mechanotransduction channel complex

The molecular identity of the transduction channel in inner ear has puzzled auditory neuroscientists for many years. Despite the rigorous investigation of various candidates, most have not met the necessary criteria to be confirmed as a true MET channel. A candidate protein must meet four well-established criteria to be recognized as a pore-forming subunit of the MET channel [5]. First, the candidate protein must be expressed at the appropriate developmental stage and localized in the right region. Considering the MET response initiation and the localization of MET channel, the candidate protein should be expressed around postnatal day ten (P10), coinciding with the time when the pore opens [6] and localized close to the basal region of tip links. Second, it should play an important role in hair cell mechanosensitivity. If its function is disrupted, MET in hair cells should be significantly affected. Third, mutations in the candidate protein should influence the mechanical response of hair cells, modifying specific properties of the MET activity. Finally, ectopic or heterologous expression of the protein in cells or lipid bilayers that are otherwise mechanically insensitive should be able to induce mechanosensitivity. This criterion is crucial to confirming that the protein functions as a structural component responsible for forming the pore of a mechanosensitive ion channel.

There are about 5000 to 27,000 hair cells within mammalian's inner ear [7,8]. This relatively small number of cells has posed challenges for using biochemical approaches to uncover the molecular constituents of the MET system. Consequently, much of our understanding of mechanotransduction has been shaped by biophysical studies, with limited insight into the molecular details. However, significant breakthroughs occurred with the identification of genes linked to deafness. Studies on these genes have revealed that hair cell mechanotransduction relies on a complex molecular machine composed of multiple components. Recent advances in structural biology have offered atomic-level views of some of these transduction molecules, enhancing our comprehension of the molecular mechanism that underpins hair cell mechanotransduction. In the following sections, we will review the known components of the hair cell MET apparatus, discuss recent research progress, and explore the roles these components play in auditory mechanotransduction.

### 3.1 TMC1/2: candidate pore-forming subunits of the MET channel complex in hair cells

TMC1 and TMC2 belong to a protein family comprising eight distinct subtypes (TMC1–8) in mammals, with homologs present in both vertebrates and invertebrates [9]. TMC orthologs have been shown to serve various functions in different species, including *Drosophila melanogaster* and *C. elegans*, and across multiple tissues. However, the connection between most TMCs and mechanotransduction remains unclear. Research in *C. elegans* suggested that TMC1 plays a developmental role, with little evidence for sensory transduction involvement [10], and demonstrated that TMC1 is essential for alkaline sensing [11,12]. Interestingly, worm TMCs (TMC-1 and TMC-2) act in neurons and muscle to promote egg laying and rhythmic Ca^2+^ activity, suggesting that TMC proteins play a role in regulating membrane excitability by mediating sodium leak currents [13]. In mouse cochlear hair cells, TMC1 has been implicated in generating a background leak conductance, which plays an important role in tuning both the frequency and intensity of sound [14]. In other species, like *Drosophila*, TMC orthologs exhibit a wide range of functional roles. For example, mutations in the *tmc* gene in fruit flies lead to defects in larval movement, which can be restored by the introduction of mammalian TMC1/2 [15]. These flies also exhibit impaired food texture discrimination, likely due to defects in detecting mechanical stimuli [10]. However, mechanosensitive currents were not detected in *Drosophila* S2 cells expressing TMC [15]. More recently, TMC4 was identified as a novel Cl^-^ channel, playing a role in perceiving high-concentration salt taste and shedding light on the involvement of Cl⁻ in salt detection [16]. Additionally, Zhang et al. [17] found that TMC6 functions as a Gαq-coupled receptor that detects noxious heat. And TMC7 was found to be co-expressed with Piezo2 in somatosensory neurons, where it acts as a regulator, suppressing Piezo2 to regulate mechanotransduction in these neurons [18].

Gene cloning research of hereditary hearing loss in humans and mice uncovered many key proteins involved in hair cell MET channel complex. Among these, TMC1 and its homolog TMC2 were discovered through studies on deaf mice and humans. In cochlear hair cells, the expressions of TMC1 and TMC2 are separated temporally during development. TMC2 is the dominant isoform during the early stages, before TMC1 takes over [19,20]. The exact reason behind developmental transition between TMC1 and TMC2 is not yet fully understood, but it is clear that TMC1 and TMC2 impart different characteristics to hair cells. The hair cells which express only TMC1 have decreased Ca^2+^ permeability and exhibit higher single-channel activity compared to those expressing only TMC2 [[20], [21], [22], [23]]. Mutations in *Tmc1* gene have been associated with both recessive and dominant forms of hearing loss [24]. Mice deficient in both *Tmc1* and *Tmc2* show no MET current in their hair cells, while *Tmc1* mutant mice retain some transducer currents in the early postnatal period [20,23]. Furthermore, Ca^2+^ permeability is modified in the *Tmc1* p.M412K knock-in mouse model, which is associated with human deafness [[20], [21], [22], [23]]. These studies indicated that TMC1/2 influence the pore properties of the cochlear hair cell MET channel.

Although a substantial portion of TMC1 is located in the endoplasmic reticulum (ER), TMC1/2 are situated in hair cells near the basal end of tip links [19]. Moreover, studies have demonstrated that TMC1/2 can interact with proteins such as PCDH15 and LHFPL5 [[25], [26], [27]]. Additionally, the positioning of TMC1 and PCDH15 in stereocilia is disrupted in the *Lhfpl5* mutant mice, further supporting the idea that TMC1/2 are components of a protein complex located near the tip links [26,28].

Biochemical assays and cryo-electron microscopy data have provided strong evidence that TMC1 is present in a dimeric form [29]. Sequence alignments have revealed that TMC1/2 share an evolutionary relationship with anoctamin (ANO)/TMEM16 family, whose members either possess Ca^2+^-activated Cl^-^ channel activities (like TMEM16A and TMEM16B) or act as Ca^2+^-activated lipid scramblases (TMEM16F) [30,31]. Homology models based on TMEM16A suggest that TMC1 shares a similar ten transmembrane (TM) domain topology [29,32]. These models also indicate the presence of a potential ion permeation pathway formed by TM4–TM7, which may constitute the pore of TMC1 [29,32]. What's more, the cryo-EM structure of the native TMC-1 mechanotransduction complex isolated from *C. elegans* confirmed a similar dimeric architecture and ten transmembrane domains [33]. However, unlike predictions for mammalian TMC1, the structure indicated that the pore is formed by TM4–TM8, highlighting the need for further structural studies.

Although homology modeling is helpful, it may not be enough to convince skeptics that TMC1 forms the pore of the MET channel. More persuasive evidence came from experiments using cysteine substitutions in TMC1 at sites near the ion permeation pore which are predicted based on the homology model [29]. By introducing these mutant constructs into hair cells that lack TMC1 and TMC2, Pan et al. [29] showed that certain cysteine mutants blocked transduction when treated with MTS reagents from the extracellular side, suggesting these residues are involved in ion permeation. Additional studies also examined TMC1 mutations associated with deafness and their impact on MET currents, further supporting the pore-forming role of TMC1 [22,[34], [35], [36], [37]].

TMC1 and TMC2 are essential for the mechanotransduction currents in hair cells, and mutations in TMC1 can alter the properties of mechanosensitive currents in mice [20,22,29,38,39]. Both TMC1 and TMC2 are expressed in hair cells and localized at the tips of stereocilia, where mechanotransduction takes place [19]. However, it remains unclear whether TMC1 and TMC2 function as ion channels themselves, and whether they can be directly gated by mechanical force. To directly record the current generated from TMC1/2, we screened various TMC1 and TMC2 orthologs from different species including humans and mice with fluorescence detection size-exclusion chromatography assay (FSEC) and identified ones that express well in heterologous cells [40]. We found that the green sea turtle's TMC1 (CmTMC1) and the budgerigar's TMC2 (MuTMC2) expressed robustly and were reconstituted into liposomes for functional studies. We removed the predicted N- and C-terminal cytoplasmic regions of the TMC plasmids to improve protein stability and yield. The proteins were subsequently expressed in Sf9 cells, purified, and reconstituted into liposomes for electrophysiological studies. Upon reconstitution into liposomes, both CmTMC1 and MuTMC2 displayed robust single-channel activity, which were validated with various MET channel blockers, including DHS, FM1–43, and neomycin. This successful reconstitution enabled us test the channel's mechanosensitivity. Upon the application of mechanical stimuli, the reconstituted TMC proteins exhibited a high sensitivity to mechanical force with high thresholds, demonstrating pronounced mechanosensitivity. Moreover, we introduced disease-causing point mutations into CmTMC1 and observed decreased or absent ion channel activity under either spontaneous or pressure-activated conditions. Although our study focuses primarily on CmTMC1 and MuTMC2, the strong evolutionary conservation of CmTMC1 and MuTMC2 with human TMC1 and TMC2 suggests that mammalian TMC1 and TMC2 are also likely to be ion channels and to be mechanosensitive. Taken together with the results of previous studies [19,20,22,29,36,38,39,41,42], the liposome reconstitution data suggest that TMC1 and TMC2 can form mechano-gated channels in vertebrates that meet most of the criteria that have been proposed, strongly supporting the link between auditory transduction and TMC1/2. However, the single-channel conductance of reconstituted CmTMC1 and MuTMC2 is 40.5 ± 2.2 pS and 35.5 ± 2.2 pS, respectively, which is smaller than the previously reported measurements for native turtle or mouse hair cell MET channels (approximately 100–300 pS) [22,23,43,44]. It is possible that the native auditory transduction channel is composed of several distinct subunits, and it is also likely that the surrounding lipids or associated proteins influence the channel's characteristics. The reasons for these effects require further investigation. Although we have successfully reconstituted turtle TMC1 and budgerigar TMC2 into the liposome system, a deeper understanding of TMC1/2′s properties require studying mammalian-derived TMC1/2 using the same approach. Furthermore, a major challenge for future research is to reconstitute the MET machinery in an exogenous expression system or liposome system. This will not only enhance our understanding of the molecular composition of the MET complex but also provide a valuable platform for investigating force-mediated gating mechanisms in greater detail.

Although TMC1 and TMC2 have been identified as promising candidate mechanotransduction channels in hair cells, controversy remains due to the lack of evidence for mechanical gating in heterologously expressed TMC1/2 proteins in cultured cells. Very recently, Fu et al. [45] addressed this longstanding issue by successfully expressing heterologous TMC1/2 proteins in the plasma membrane, providing direct evidence that mammalian TMC1/2 function as mechanotransduction channels in hair cells. To overcome the trafficking challenge, the authors leveraged the OSCA/TMEM63 superfamily, which is localized to the plasma membrane and shares topological and structural similarities with TMC1/2 [33,[46], [47], [48]]. They generated chimeric constructs of mouse TMC1/2 and OSCA1.1 by replacing the putative pore region (TM3–7) of mouse TMC1/2 with the corresponding structural region of OSCA1.1. This domain-swapping approach successfully facilitated the trafficking of mouse TMC1/2 to the plasma membrane in HEK293T cells. Subsequent alanine mutation screening of the TM3 region identified several point mutations at both ends of the transmembrane segment that enabled membrane expression and mechanical activation. Pharmacological analysis with mechanotransduction blockers and I-V characterization revealed that mouse TMC1 (H353A) and TMC2 (H406A) function as mechanically gated ion channels. To investigate whether wild-type, full-length human TMC1/2 are mechanically gated when expressed in the plasma membrane, the authors employed a whole-genome CRISPRi screening strategy, identifying 93 genes that promote the surface expression of wild-type human TMC2. Flow cytometry and non-permeabilized staining showed that knockdown of ARL1, RGP1, and UROD enhanced TMC1/2 trafficking to the plasma membrane in HEK293T cells. Electrophysiological assays demonstrated that wild-type human TMC1/2 (co-expressed with shUROD in Piezo1-KO HEK293T cells) responded robustly to mechanical poking, displayed stretch-activated currents, and exhibited clear single-channel activity. Deafness-associated mutations in human TMC1 altered reversal potentials, further supporting the role of TMC1/2 as pore-forming subunits of mechanically gated ion channels. The successful expression of TMC1/2 in heterologous cell membranes marks a significant advancement, enabling detailed studies of TMC1/2 gating mechanisms, their interactions with other MET complex components, and their role in transducing mechanical stimuli. These findings open new avenues for research and facilitate the development of therapeutic compounds targeting human TMC1/2 for the treatment of auditory disorders.

### 3.2 TMIE: regulator of the pore and gating characteristics of MET channel

TMIE is also a crucial component of the MET channel in cochlear hair cells [49]. TMIE was also identified via cloning deafness genes in humans (DFNB6) and mice (the *spinner* mouse model), and it is predicted to have two TMs [50,51]. TMIE specifically binds to TMC1/2, and disruptions to this binding prevent mechanotransduction. Deletions in the N-terminal region of TMIE also alter the channel's response to mechanical stimuli [52]. In absence of TMIE, TMC1/2 cannot form functional MET channels in cochlear hair cells. Moreover, TMIE also binds to other key components, including LHFPL5 and PCDH15 at the lower end of the tip links [49,52]. In zebrafish, TMIE is involved in regulating TMC localization [53]. In *Tmie* null mutation mouse model, the hair cells mechanotransduction is abolished [52]. Interestingly, overexpressing TMC1/2 into hair cells of *Tmie* mutant mice relocates TMC1/2 back into the stereocilia, but this does not restore MET currents. Despite these insights, the precise mechanism through which TMIE influences the cochlear hair cells channel pore properties is still not fully understood. The native cryo-EM structure of *C. elegans* TMC-1 channel complex has shown that TMIE comprises a single-pass transmembrane helix, connected via an elbow-like linker and a cytoplasmic helix. The interaction between TMC-1 and TMIE is primarily mediated by TMIE's cytosolic elbow. Conserved arginine residues, R49 and R52, in TMIE form hydrogen bonds with the backbone carbonyl atoms in the TM6 and TM8 helices of TMC-1. These interactions are vital, as corresponding mutations in human residues (R81C and R84W) are associated with hereditary deafness. This highlights the vital role of these hydrogen bonds in stabilizing the TMC-1 and TMIE complex. Further stabilization is provided by hydrophobic interactions between nonpolar residues in the TMIE elbow and TM6 of TMC-1. Within the membrane, TMIE and TMC-1 create an intramembrane cavity that is filled with lipids, which interact hydrophobically with both TMIE and the pore-forming helices of TMC-1. These lipids act as a bridge between the two subunits, playing a role in the complex's stability and functionality. Moreover, TMIE is palmitoylated at cysteine 44, located at the cytoplasmic boundary of its transmembrane domain, and this modification is thought to affect its interaction with TMC-1 by extending an acyl chain along TM8. Due to its proximity to the TMC-1 pore and lipid-binding regions, TMIE likely contributes to the gating of the MET channel by sensing membrane tension to regulate the channel's activity. These findings underscore TMIE's crucial role in mechanotransduction and its significance in auditory function [33].

Genetic research in humans further underscores the importance of TMIE as a key subunit in MET channel complex. Several point mutations associated with hearing loss have been discovered in the cytoplasmic C-terminal region of TMIE [50]. These alterations are located within a polar region interacting with both TMC1 and phospholipids, notably PIP2 [52]. Such mutations disrupt the interaction with PIP2, consequently modifying the pore characteristics of the MET channel. This results in lower single-channel activity and altered Ca²⁺ selectivity [52].

### 3.3 CIB2 and CIB3: modulating TMC1/2 localization within hair cells

CIB2/3 are small proteins (∼22 kDa) belonging to members of the calcium- and integrin-binding proteins, which includes four members (CIB1–4) in mammals. These proteins possess multiple EF-hand domains, facilitate Ca^2+^ binding [54]. Genetic mutations in CIB2 have been linked to nonsyndromic deafness (DFNB48) and Usher syndrome type 1 J, highlighting its critical role in auditory function. Null mutations in *Cib2*, as well as specific point mutations associated with deafness, impair mechanotransduction and cause hair bundles degeneration in cochlear hair cells [43]. Studies have shown that CIB2 interacts with TMC1/2, playing a critical role in the MET process of hair cells [43,55,56]. Notably, CIB3 has the ability to offset the absence of CIB2, especially within vestibular hair cells, suggesting functional redundancy that helps maintain mechanotransduction even when CIB2 is absent or mutated. This redundancy preserves the essential sensory functions of these cells.

Biophysical studies have demonstrated that TMC1 binds to CIB2/CIB3 via two cytosolic regions (amino acids 81–130, amino acids 303–347) [43,55]. To better understand this interaction, Liang et al. [55] co-expressed CIB3 with a fragment of TMC1 that includes the first cytosolic loop (amino acids 298–352) and resolved the complex's crystal structure. The high-resolution X-ray crystallographic structure of the TMC1-CIB3 complex identified a hydrophobic groove in CIB2/3 that facilitates the binding to TMC1/2, resembling the interaction observed between Kv4 and KChIP1. Further molecular modeling predicts that TMC1/2 interact with CIB2 in a way that mirrors their engagement with CIB3. This hypothesis is reinforced by cryo-EM structural data of the native TMC-1 channel complex, showing that TMC1 also binds to CALM1, the worm's ortholog of CIB2/3 [33]. The cryo-EM structure further demonstrated that CALM1 covers the cytoplasmic surface of the ion channel, with TMC1’s first cytoplasmic loop forming long and short α-helices that fit into a groove on CALM1, suggesting a conserved interaction mechanism across different species.

The precise way in which CIB2 modulates MET channel activity is still not fully understood. Ca²⁺ plays a particularly intriguing role in MET gating, both affecting both extracellular calcium homeostasis and intracellular calcium-dependent adaptation mechanisms [[57], [58], [59]]. Extracellular Ca²⁺ is indispensable for maintaining the structural integrity and functionality of the MET channel, while intracellular Ca²⁺ is involved in feedback mechanisms that regulate channel sensitivity. These processes fine-tune the hair cells’ response to mechanical stimuli, ensuring accurate signal transduction and adaptation to changing sound levels. NMR studies have shown that the structure of CIB2 is responsive to alterations in Ca²⁺ concentrations [60]. Additionally, recent findings indicate that CIB2/3 functions as a calcium sensor in MET, where the TMC1-CIB2/3 complex undergoes a calcium-triggered conformational shift, emphasizing the critical role of calcium in MET channel function [61]. This implies a dynamic regulatory mechanism in which Ca²⁺ influx directly affects the molecular interactions necessary for mechanotransduction.

### 3.4 The tetraspan LHFPL5: an essential component for transmitting tension in the tip link to activate the MET channel

LHFPL5 (also referred to as TMHS) is a membrane protein with four predicted transmembrane helices and is a member of the tetraspan protein superfamily. It was first identified in the *hurry-scurry* (*hscy*) mutant mouse model, which exhibits deafness and vestibular dysfunction [62]. Mutations in *LHFPL5* gene in humans have been linked to autosomal recessive (AR) nonsyndromic hearing loss (ARNSHL), classified as DFNB67 [63,64]. In mice, *Lhfpl5* is expressed as MET currents begin to develop, with its expression persisting into adulthood. LHFPL5 reaches peak expression during P3–P6 and is localized at the tips of all stereocilia rows [28,65]. Previous studies have indicated that PCDH15 binds to TMC1, moreover, biochemical and cryo-EM structural studies have shown that PCDH15 also binds to LHFPL5 at its TM and cytoplasmic domains (Fig. 4) [25,26,28,49,66,67]. These findings support the hypothesis that force in the tip links is conveyed to the MET machinery via the interaction between LHFPL5 and PCDH15, which is subsequently coupled to TMC1 [25,28,67].

While LHFPL5 was previously thought to be essential for correctly positioning TMC1 at the transduction site located at stereocilia tips, it is not essential for the formation of an ion channel [26]. In *Lhfpl5* knockout mice, the levels of TMC1/2 in stereocilia are reduced, and although the MET current also reduced, it remains detectable (200–400 pA) in neonatal OHCs [68], suggesting a reduction in mechanosensitivity of the channel's gating. In zebrafish, *Lhfpl5* deficiency does not affect the localization of TMC1, and TMCs remain expressed in the hair cell stereocilia in *Lhfpl5* mutant lines [69]. The reduced MET current observed in *Lhfpl5* knockouts may result from the stabilizing role of LHFPL5 in the MET complex. Recent study has demonstrated that the mechanosensitivity of the channel's gating in *Lhfpl5* knockouts was reduced several-fold, and the gating stiffness was almost entirely eliminated [68]. These recent findings, along with previous research [28,67,70], suggest that LHFPL5 enhances the hair cell mechanosensitivity by linking PCDH15 to TMC1, establishing LHFPL5 as the key mediator of transmitting force to the hair cell mechanotransduction channel.

### 3.5 PCDH15 and CDH23: molecular components of the tip link

PCDH15 and CDH23 are both members of the cadherin superfamily, and they are among the earliest molecules to be definitively associated with the mechanotransduction apparatus in cochlear hair cells, as revealed through genetic research. Variants in *PCDH15* and *CDH23* have been linked to hearing impairment [[71], [72], [73]]. Since the discovery of tip links, they have been recognized as the structures responsible for transmitting mechanical force to the MET channels [74]. The integrity of tip links is critical for mechanotransduction, and mutations in *Pcdh15* or *Cdh23* genes lead to disrupted MET currents [[75], [76], [77]]. Subsequent studies established that these proteins are key components of the tip links, which had been discovered in 1984 through ultrastructural observations of hair bundles [74,[78], [79], [80], [81]]. Immunohistochemistry and biochemical studies further demonstrated that PCDH15 and CDH23 are located at the upper and lower regions of the tip links, respectively. The N-terminal regions of cadherins align along the tip link filaments, with CDH23 homodimers engaging *in trans* with PCDH15 homodimers, forming the upper and lower sections of the tip links [78]. Both PCDH15 and CDH23 contain multiple extracellular cadherin (EC) repeats—11 and 27, respectively. Together, these repeats form an extracellular filament with a total of 76 EC domains, spanning an impressive length of approximately 150 nm [78]. Structural studies using crystallography have shown that the interaction between PCDH15 and CDH23 occurs through *trans*-interactions facilitated by their cadherin repeats, which confirm the characteristic cadherin fold in their EC domains [[82], [83], [84], [85], [86], [87], [88], [89], [90], [91]] (Fig. 4). Parallel structural studies of PCDH15 revealed that the dimerization sites are positioned at the EC3 domains of two PCDH15 molecules, with another dimerization site located nearer to the cell membrane [66,85].

Early *in vitro* biochemical assays suggested that PCDH15 interacts with TMC1 [27,36]. However, a later structural study did not confirm this interaction [66]. Instead, it was found that PCDH15 forms a stable complex with LHFPL5. The resolved cryo-EM structure of the PCDH15/LHFPL5 complex demonstrated that dimeric PCDH15 pairs with two LHFPL5 molecules in a symmetric manner [66] (Fig. 4). Extensive interactions between the TM domains of both proteins facilitate the assembly of the PCDH15/LHFPL5 complex. These two LHFPL5 proteins act as stabilizers, or clamps, holding the transmembrane domains of dimeric PCDH15 in place. This indicates that mechanical force from the tip link could be relayed to the MET channel via LHFPL5. Further structural and functional research is required to fully elucidate how force is transmitted to the MET channel from the tip links and how this process leads to the opening of the channel pores.

## 4 Additional components critical for hair cellmechanotransduction

While core components such as TMC1 and TMC2 are directly involved in forming the mechanotransduction channel, many other molecules are essential to ensure this process occurs efficiently and reliably. The complexity of the cochlear environment, along with the mechanical demands on hair cells and the need for precise regulation of MET activity, requires additional proteins to maintain the stability, localization, and modulation of the MET complex. These accessory proteins help preserve the integrity of the mechanosensitive response. Several have been identified as essential for proper functioning of the MET machinery.

Harmonin, SANS and MYO7A, located at the upper end of the tip link, play roles in regulating hair cell mechanotransduction. These proteins may influence the function of tip link components or modulate hair bundle stiffness, affecting the sensitivity and responsiveness of the MET channel [[92], [93], [94], [95], [96], [97], [98]]. Besides proteins that directly participate in the MET process, several others play a role in the development of hair bundles, a critical factor for effective mechanotransduction. Whirlin, MYO15A, and CLRN1/2 are key players in both the development and maintenance of hair bundles, the structures hosting the MET apparatus [[99], [100], [101]]. Although these proteins are not part of the MET channel, they are essential for maintaining the structural integrity and functional performance of hair bundles, thus indirectly facilitating efficient mechanotransduction. Malfunctions in these proteins can lead to secondary effects on MET, resulting in hearing loss.

Other proteins, such as TOMT, LOXHD1, and PIP2, have been directly linked to the MET machinery. TOMT (also referred to as COMT2 or LRTOMT in humans) is involved in the assembly and trafficking of the MET machinery. Although TOMT is not directly part of the MET channel, it is crucial for ensuring that MET components are correctly localized to the stereocilia [102]. Mutations in *TOMT* can lead to defects in MET assembly and function, contributing to hearing impairment [[102], [103], [104]]. LOXHD1 is another key protein, essential for preserving the structural integrity of stereocilia [105]. While not part of the MET channel itself, LOXHD1 is necessary for the function and stability of hair bundles. LOXHD1 specifically interacts with TMC1, CIB2, LHFPL5, and PCDH15. A latest study demonstrated that LOXHD1 plays a crucial role in anchoring TMC1-pore forming subunits to the tip link, and indicated that mature auditory channels driven by TMC1 rely on LOXHD1 to remain connected to the tip link and maintain functionality [106]. Mutations in LOXHD1 are linked to progressive hearing loss, likely due to its role in maintaining the necessary structural elements for MET functionality [107,108]. PIP2, a type of phospholipid, is critical for regulating ion channel activity, including that of the MET channel in hair cells. PIP2 is believed to affect the gating of the MET channel, and its interaction with other MET components is likely essential for efficient transduction of mechanical signals. Changes in PIP2 levels or its interaction with the MET machinery could disrupt mechanotransduction and result in hearing loss [109,110].

In a recent paper, Lee et al. [111] proposed that the Piezo1/2 constitute part of the MET complex in the mammalian inner ear hair cells and proposed the MET complex includes multimers of the Piezo channel subunits that serve along with other regulatory and indispensable binding partners such as TMC1 to confer a functional MET unit. Piezo1/2 proteins, while playing crucial roles in mechanotransduction in other systems such as touch and proprioception, do not meet the essential criteria to be considered part of the hair cell mechanotransduction complex [112]. Briefly, first, the timing and spatial expression of Piezo1/2 proteins in auditory hair cells are not aligned with the requirements. Although Piezo2 is transiently expressed during the first postnatal week, its expression is low and rapidly declines [113]. Furthermore, Piezo1 expression has not been detected in the inner ear, and the low expression of Piezo2 detected in hair cells is insufficient to support its role in mechanotransduction. Secondly, the localization of Piezo1/2 proteins is problematic. Unlike TMC1/2 and other transduction molecules, which are clearly localized to the tips of the stereocilia, Piezo 2 is found on the apical surface of the hair cell, not at the tips where mechanotransduction occurs. Lee et al. also reported that when Piezo1 and Piezo2 were tagged and expressed in hair cells, the proteins were diffusely distributed in the hair bundle and did not concentrate at the tips of stereocilia, where mechanotransduction occurs. This mislocalization further challenges the hypothesis that Piezo proteins play a central role in mechanotransduction in hair cells. Moreover, genetic deletion of Piezo1 and Piezo2 does not result in a loss of sensory transduction. The conditional deletion of Piezo2 did not affect conventional hair cell transduction currents [113,114], further supporting those Piezo proteins are not essential for the primary mechanotransduction process. Lee et al. [111] generated mice conditionally deficient in Piezo1 and Piezo2 but did not present transduction currents recorded from those mice. Finally, although mutations in Piezo2 can lead to abnormal mechanotransduction currents, these changes do not affect the primary transduction currents nor the ion channel properties [111,113]. These findings suggest that while Piezo2 might influence some mechanosensitive responses, it does not directly participate in the core mechanotransduction process in auditory hair cells.

Identifying and characterizing the full array of proteins involved in mechanotransduction remains a significant challenge for auditory neuroscientists. While there is now abundant and rigorous evidence supporting the molecular identity of 8 to 10 different protein components of the hair cell MET complex (Fig. 5), the roles and interactions of other proteins within the MET complex are still being explored. The complexity of the cochlear environment and the precise timing and localization needed for proper MET function underscore the importance of these additional molecules.

## 5 Molecular assembly model and gating mechanism of MET channel complex

**Fig. 3:**
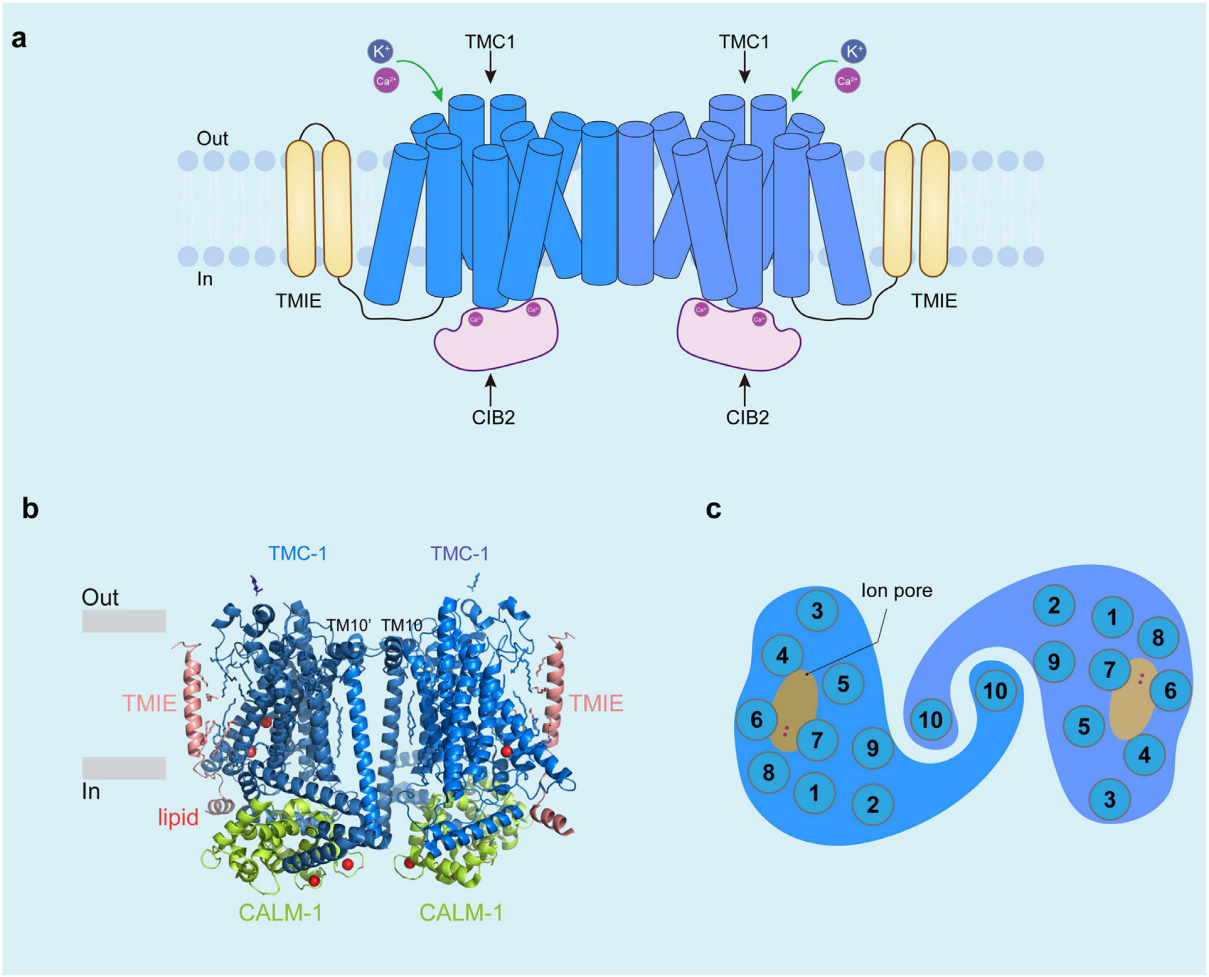
**Structural representation of the MET complex.** (a) Schematic of the MET complex based on the native cryo-EM structural data from *C. elegans*, illustrating the key components involved in ion conduction. The TMC1 protein forms the central ion channel through which K⁺ and Ca²⁺ ions flow, enabling depolarization of the hair cell. TMIE is shown as a supporting transmembrane protein, anchoring the channel, while CIB2 interacts with the intracellular side of TMC1 to regulate channel function. (b) Structural model of the native TMC1 complex from *C. elegans*, highlighting the arrangement of two TMC1 subunits (dark and light blue) within the lipid bilayer. TMIE (salmon) is positioned alongside TMC1 to stabilize the structure. CALM-1 (green) is depicted at the intracellular surface, possibly modulating TMC1 activity. Lipid molecules are shown interacting with the channel, contributing to its structural stability. (c) Top-down view of the TMC1 ion pore, with the arrangement of the ten transmembrane helices labeled. The helices form a channel through which ions pass, with specific residues creating a selective ion pore to regulate the flow of K⁺ and Ca²⁺ ions.

The content summarized and discussed above reveals an assembly model of the hair cell MET channel complex, which is a symmetrical dimeric structure containing two pores, with each monomer consisting of TMC1, TMIE, and CIB2 molecules (Fig. 3). The most direct evidence comes from the cryo-EM structure of the native *C. elegans* TMC1 complex. Two main models have been proposed for MET channel activation: the tethered-channel model, which argues that MET channel opening is regulated by the mechanical tethering of transmembrane proteins, and the lateral-tension model, which suggests that the activation of these channels is driven by mechanical tension on the membrane. The tethered-channel model proposes that the mechanical force on the tip links causes a physical interaction with the MET channels, directly leading to their opening, and that the MET channel complex is tethered to the tip link via LHFPL5, although no structural biological data are available to support this model. Specifically, the MET channels are anchored to extracellular tip links, and to the intracellular cytoskeleton. When sound vibrations cause deflection of the stereocilia, the tip links are stretched, generating mechanical forces. These forces are transmitted through protein–protein interactions between the tip links and the MET channels. This mechanical stretch directly causes the channels to open, allowing the influx of ions into the hair cell. The tethered-channel model emphasizes the crucial role of mechanical tethering and protein interactions in the activation of MET channels. The discovery of the TMC-1/CIB/ankyrin functional triple complex in *C. elegans* [115] offers compelling support for the tethered-channel model and introduces the intriguing possibility that ankyrin proteins may serve as the long-awaited gating spring. In the lateral-tension model, MET channels are freely located within the membrane at the tips of stereocilia, without being tethered to the tip links or the intracellular cytoskeleton. In this model, the opening of the channels is driven by membrane tension, which is generated when the tip links are pulled during stereocilia deflection. The forces exerted on the tip links create tension in the membrane, which is sufficient to directly activate the MET channels. There is no direct physical connection between the channels and the tip links or the cytoskeleton; instead, the membrane tension itself is proposed to serve as the gating mechanism, emphasizing the role of membrane mechanics in MET channel activation. Our previous work [40] provides direct evidence supporting this model. When truncated TMC1 and TMC2 proteins from turtle and budgerigar were reconstituted into liposomes, they appeared to be activated upon the application of negative pressure to the membrane. Perhaps the activation mechanism of the MET channel in hair cells cannot be fully explained by either the tethered channel model or the lateral-tension model alone. Both mechanisms might cooperate to establish the exquisite force sensitivity of this channel. Given this and in light of recent research findings, Zheng and Holt [116] reconciled the two models and proposed a grand unifying model of hair cell mechanotransduction (Fig. 6). In the new model, TMC1 interacts with TMIE and is anchored to the actin cytoskeleton via CIB2/ankyrin. The TMC1/TMIE complex is situated in the membrane area surrounding the PCDH15/LHFPL5 complex, but there is no direct physical link between them. When the hair bundle is deflected, the tip membrane of the shorter stereocilia is pulled away from the underlying cytoskeleton by the tension in the tip links. This mechanical strain activates TMC1 through membrane deformation and the participation of ankyrin proteins.

**Fig. 4:**
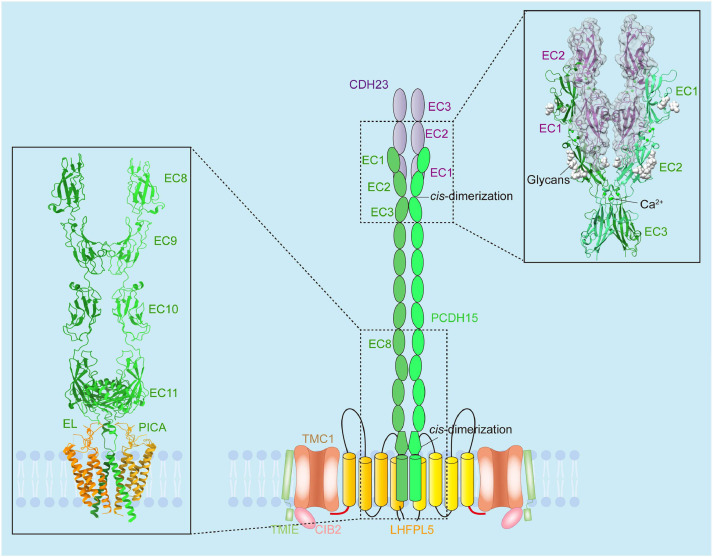
**Structure of the LHFPL15-PCDH15 complex and tip link complex in auditory hair cells.** This illustration shows the molecular interactions involved in forming the tip link and the binding between PCDH15 and LHFPL5. The tip link is established through interactions between CDH23 (purple) and PCDH15 (green), which play a crucial role in converting mechanical forces from stereocilia deflection into cellular signals. Each PCDH15 molecule includes 11 extracellular cadherin (EC) domains (EC1–EC11) and a PICA (interacting-channel associated) domain. EC1 and EC2 participate in a “handshake” bond with a CDH23 dimer. Additionally, PCDH15 forms dimers via intermolecular interactions at EC3 and the PICA domain. Right Inset: The model shows the interaction of PCDH15 EC1-EC3 with CDH23 EC1-EC2. This was created by aligning PCDH15 EC1-EC2 from the PCDH15 EC1-EC2 structure (PDB: 4APX) to the PCDH15 EC1-EC3 *cis*-dimer structure (PDB: 6CV7) [85]. Left Inset: Structural model of the PCDH15 (EC8-EC11)/LHFPL5 complex (PDB: 6C14) [66].

**Fig. 5:**
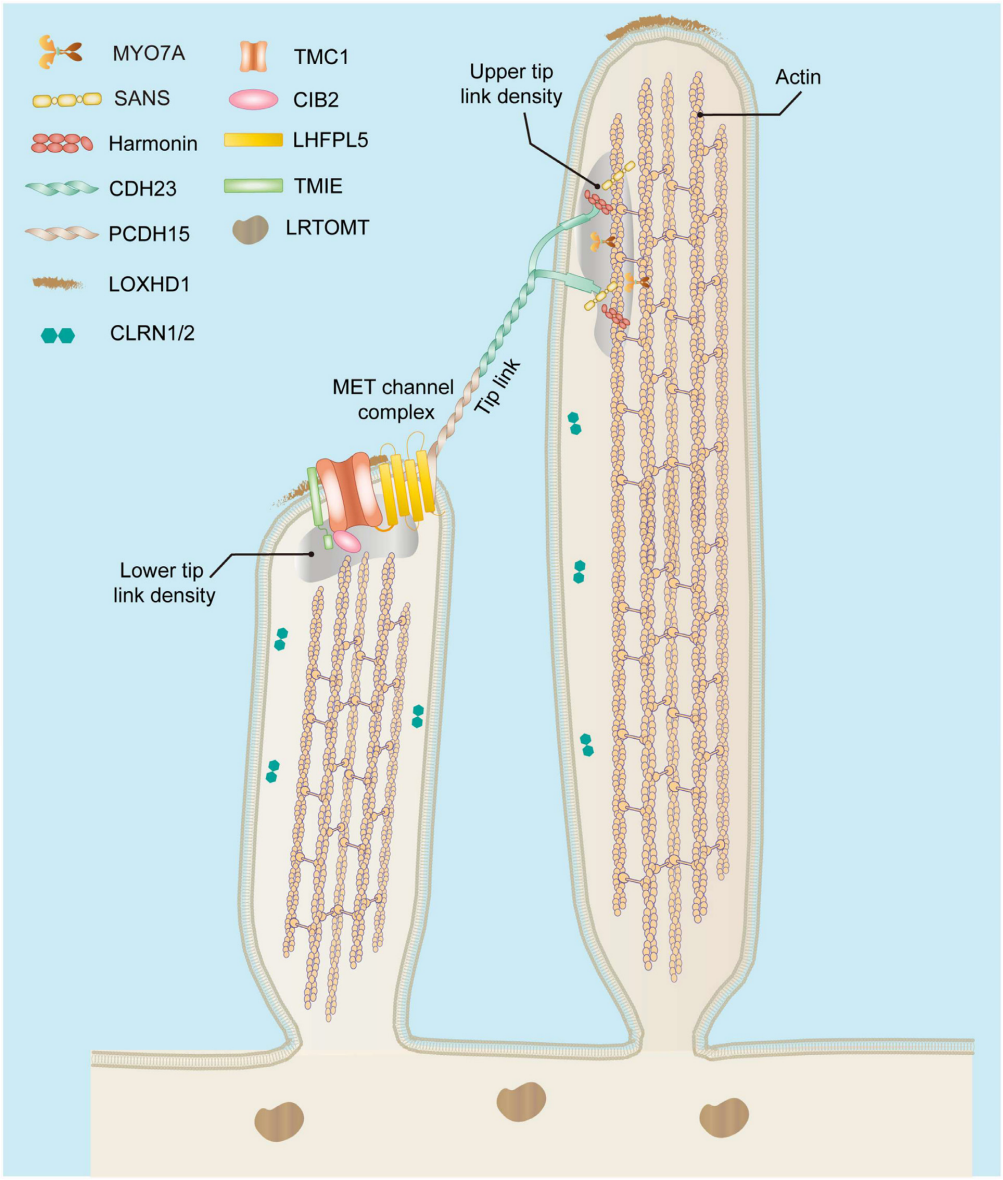
**Molecular composition and structural organization of stereocilia in auditory hair cells.** This figure illustrates the molecular architecture of stereocilia within auditory hair cells, focusing on the localization of key proteins involved in mechanotransduction and structural integrity.

**Fig. 6:**
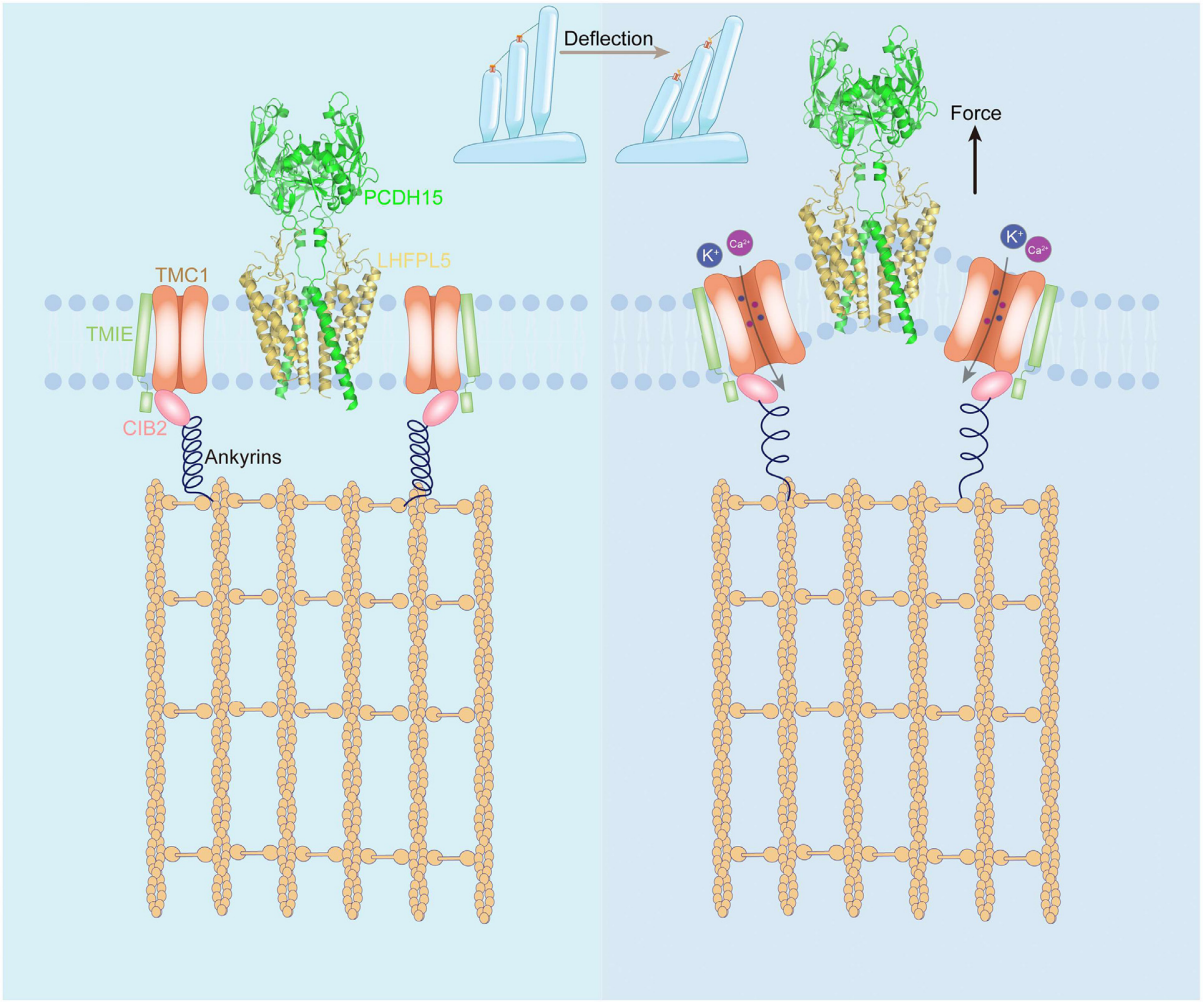
**Proposed working model of hair cell mechanotransduction.** TMC1 interacts with TMIE and is anchored to the actin cytoskeleton via CIB2/ankyrin. The TMC1/TMIE complex is situated in the membrane area surrounding the PCDH15/LHFPL5 complex, but there is no direct physical link between them. When the hair bundle is deflected, the tip membrane of the shorter stereocilia is pulled away from the underlying cytoskeleton by the tension in the tip links. This mechanical strain activates TMC1 through membrane deformation and the participation of ankyrin proteins.

## 6 Genetic mutations impacting mechanotransduction and their role in hearing loss

Hearing loss is one of the most prevalent sensory impairments, with roughly half of all congenital cases attributed to genetic causes. So far, variants in >150 genes have been linked to nonsyndromic hereditary hearing loss, while over 400 genetic syndromes also affect auditory function (https://hereditaryhearingloss.org/). Many of these mutations specifically target cochlear hair cells, although the underlying mechanisms remain partially unclear. In particular, mutations that disrupt the MET machinery are significant contributors to hearing loss [73]. These genetic changes compromise hair cells’ ability to perform their essential role in hearing, leading to various types of hearing impairment. Understanding these mutations is crucial for developing targeted therapies to prevent or mitigate hearing loss associated with MET dysfunction. Mutations have been identified in the genes encoding all known component of the MET machinery. Mutations in some genes can directly affect the function of MET components, while some indirectly influence the function and properties of MET by impacting the development and survival of cochlear hair cells or by affecting hair bundle morphogenesis. In the following sections, we will primarily discuss the gene mutations that have a direct impact on the MET machinery's function (Table 1).

**Table 1:** **Auditory hair cell mechano-electrical transduction-related deafness genes**.

Gene	Chromosomal Location	Disease (OMIM)	Inheritance Mode	Onset	Auditory Phenotype	Protein Localization	Molecular Function	Pathogenesis Mechanism	Research/Therapeutic Insights	References
*TMC1*	9q21.13	DFNB7/11 (600974)	AR	Congenital	Severe-to-profound deafness	Found in MET channels within stereocilia	Pore-forming subunit of MET channel, allowing ion influx in response to sound-induced hair bundle deflections, which is signal initiation.	Loss of MET channel integrity in stereocilia, affecting the cellular tension required for normal sound transduction.	Advanced research into *tmc1* gene therapy uses precise gene-editing tools to restore auditory function by enhancing MET channel gating properties.	[42,[130], [131], [132]]
		DFNA36 (606705)	AD	Postlingual	moderate to severe deafness				*in vivo* delivery of genome editing agents.	
*LHFPL5*	6p21.31	DFNB67 (609427)	AR	Congenital	Severe-to-profound deafness	Localized at the insertion lower tip link in hair cells	Establish maximal force sensitivity of the MET channel; A key element in force transmission from the tip link to the MET channel.	Lower tip link insertion impairment, reducing met channel stability and leading to gradual hearing loss in cochlear cells.	*Lhfpl5*-targeted treatments explore stabilizing MET channels via small molecules, and early gene therapy shows potential in congenital hearing loss models.	[[62], [63], [64],^133^]
*TMIE*	3p21.31	DFNB6 (600971)	AR	Congenital	Severe-to-profound deafness	Anchored within the cytoskeletal stereocilia	An essential component of MET channel complex; affect the pore properties of MET channel.	Ankyrin-based cytoskeletal disruption, impacting hair bundle stiffness and reducing auditory sensitivity.	Research centers on gene therapy approaches aimed at restoring TMIE function in auditory hair cells to enhance mechanotransduction channel stability and improve hearing outcomes.	[[49], [50], [51], [52]]
*CIB2*	15q25.1	DFNB48 (609439)	AR	Congenital	Severe-to-profound deafness	Embedded within the hair cell MET complex	Regulates MET channel gating by modulating the opening and closing of the transduction channels in response to stereocilia deflection.	Affect MET channel function, resulting in reduced transduction efficiency in hair cells.	Gene therapy and molecular strategies to restore CIB2 function in hair cells, aiming to stabilize MET channels and improve sensory cell resilience.	[43,56,117,134]
*PCDH15*	10q21.1	DFNB23 (609533)	AR	Prelingual	Severe-to-profound deafness	Lower region of the tip link in cochlear hair cells	Serves as a component of the tip link structure, crucial for transmitting sound-induced forces between stereocilia in hair cells.	Loss of MET current in auditory hair cells due to tip link disruption, resulting in impaired auditory signaling.	Gene therapy approaches show promise in restoring tip-link integrity to address deafness associated with *Pcdh15* mutations.	[72,135,136]
		USH1F syndrome (602083)	AR	Congenital	Severe-to-profound deafness	Kinociliary links and lateral links within the hair cell bundle	Maintains cohesion of the hair bundle, allowing stereocilia to work together as a unit for effective MET under sound stimuli.	Disruption of hair bundle morphology, impacting hair cell cohesion and leading to severe hearing loss.	Gene replacement therapies for USH1F-related deafness and vision loss, targeting dual sensory recovery through crispr and viral vectors.	
*CDH23*	3p25.3, 10q22.1	DFNB12 (601386)	AR	Prelingual	Moderate-to-profound deafness	Upper region of the tip link	Acts as an upper anchor for the tip link, connecting adjacent stereocilia and facilitating precise force transmission during sound detection.	Loss of MET current due to upper tip link malfunction, causing progressive damage to hair cell structure and function.	Explore cochlear gene delivery systems and CRISPR-based editing to restore function in moderate-to-profound deafness cases.	[71,118,121]
	10q22.1	USH1D syndrome (601067)	AR	Congenital	Severe-to-profound deafness	Kinociliary links and lateral links	Essential for the cohesion and stability of the hair cell bundle, ensuring organized stereocilia movements critical to mechanosensitivity.	Defective hair bundle morphogenesis, leading to disorganized stereocilia structure and significant auditory deficits.	Emerging therapeutic efforts for *USH1D* aim to repair kinociliary and lateral links, with strategies that combine gene therapy with sensory cell regeneration.	
*USH1C/ Harmonin*	11p15.1	USH1C syndrome (276904)	AR	Congenital	Progressive postlingual deafness	Associated with actin filaments in stereocilia	Provides structural support to actin filaments, ensuring stereocilia stability necessary for reliable mechanotransduction.	Dysfunctional harmonin protein affects the stability and organization of stereocilia in auditory and vestibular hair cells, and leads to progressive photoreceptor degeneration.	Gene therapy and RNA-based strategies to correct mutations in *USH1C*, aiming to restore both hearing and vision by targeting the function of harmonin in sensory cells.	[93,[122], [123], [124], [125],^137^]
		DFNB18A (602092)	AR	Prelingual	Severe-to-profound deafness	Localized in the nucleus	Regulates transcription of genes related to inner ear development, influencing hair cell differentiation and auditory system maturation.	Loss of MET current, dysmorphic stereocilia	Gene therapy and RNA-based treatments to restore harmonin function, aiming to improve hearing and balance by stabilizing hair cell structure and function in the inner ear.	
		DFNA (?)	AD	Postlingual	Moderate-to-severe deafness	Found along the stereocilia membrane	Supports stereocilia linkage stability, ensuring hair cell resistance to mechanical wear and tear during sound exposure.	Disruption of the interaction between CDH23 and harmonin; Reduced MET current.		
*USH1G/ SANS*	17q25.1	USH1G syndrome (606943)	AR	Congenital	Severe-to-profound deafness	Concentrated within stereocilia bundles	Maintains proper bundle morphology in hair cells, aiding in consistent mechanotransduction performance and sound sensitivity.	Disorganization and instability of hair cell stereocilia; loss of MET current	Gene therapy approaches to restore the function of the SANS protein, crucial for maintaining the structural integrity of sensory hair cells, with the goal of improving hearing and balance.	[98,138,139]
*LRTOMT/COMT2*	11q13.4	DFNB63 (611451)	AR	Congenital	Severe-to-profound deafness	Distributed along stereocilia	Bind to putative components of the MET channel and is essential for the transport of some of these components into the mechanically sensitive stereocilia of hair cells.	Loss of MET current, trafficking defect in MET machinery	Gene replacement therapy aimed at restoring LRTOMT function to improve auditory hair cell mechanotransduction and support hearing restoration.	[[102], [103], [104]]
*LOXHD1*	18q21.1	DFNB77 (613079)	AR	Postlingual (7–8 years), rapidly progressive	Mild-to-moderate	Located in hair cell stereocilia	Indispensable for maintaining TMC1 auditory mechanosensitive channels at the site of force transmission	Loss of MET current after the first week	Gene therapy strategies to restore LOXHD1 function, aiming to enhance mechanotransduction and protect auditory hair cells to improve hearing.	[107,108]
*CLRN1*	3q25.1	USH3A syndrome (276902)	AR	Postlingual	Moderate-to-severe deafness	Hair bundle stereocilia	Stereocilia development, presynaptic maturation	Disruption of hair cells structure and function; progressive degeneration of auditory and visual cells.	Target gene therapy and small molecule treatments to restore CLRN1 function, aiming to preserve both hearing and vision by supporting the integrity of sensory cells in the inner ear and retina.	[99,126,140]
*CLRN2*	4p15.32	DFNB117 (619174)	AR	early childhood	Moderate-to-profound sensorineural deafness	Hair bundle stereocilia	Maintenance of stereocilia integrity	Affect the stability and function of auditory hair cell synapses, leading to impaired mechanotransduction and progressive hearing loss.	Gene therapy approaches to correct mutations affecting the MET pathway, aiming to restore auditory function by enhancing mechanotransduction in hair cells.	[101,127]

AR, autosomal recessive; AD, autosomal dominant; ?, uncertain.

Variants in MET-related genes can be inherited in either recessive or dominant patterns, resulting in nonsyndromic or syndromic deafness (Table 1). Interestingly, different mutations within the same gene can lead to varying disease manifestations (Table 1). For instance, variants in the *TMC1* gene are linked to autosomal dominant (AD) nonsyndromic hearing loss, referred to as DFNA36 [42]. Numerous *TMC1* missense mutation studies have been discovered in individuals with DFNA36, Such as *TMC1* p.G417R, p.M418K, p.T422K, p.D572N, and p.D572H. The pathogenic mechanisms and functional effects of these mutations have been studied by generating various mutant mouse models. The *Tmc1* p.M412K mutation mouse model (the mutate site corresponding to *TMC1* p.M418K), known as Beethoven (*Bth*) mouse, replicates the DFNA36 phenotype, displaying reduced calcium permeability in the MET channel, likely caused by a dominant-negative effect [22,26]. Additionally, the *TMC1* p.D572N mutation leads to the expression of TMC1 unstable by affecting its interaction with LHFPL5 [25]. Furthermore, mice carrying the *Tmc1* p.T416K mutation (analogous to *TMC1* p.T422K) exhibit reduced resting channel open probability (Po) and Ca^2+^ permeability, without changes to single-channel conductance [34].

Variants in *TMC1* are also linked to ARNSHL, specifically DFNB7/11 [42]. DFNB7/11 can arise from various types of mutations in *TMC1*, including nonsense, splicing, frameshift, and missense mutations. The characteristic phenotype of this mouse model is the complete loss of mechanotransduction function in hair cells, similar to what is observed in *dn* mice [20].

The *Tmc1* p.D528N mutation, which is inherited in recessive mode, leads to significant reduced (7-fold) Ca^2+^ permeability and largely reduction (of 37%) unitary conductance in MET current, suggesting that this mutation affects the pore properties of TMC1 based on the modeling structure [32,34]. Several *TMC1* mutations associated with DFNB7/11 are likely to decrease or eliminate MET current and impair Ca^2+^ permeability, causing severe deafness.

Variants in *TMIE* and *CIB2* have been linked to DFNB6 and DFNB48, respectively [50,117]. These genetic alterations interfere with key components of the MET system, resulting in a loss of mechanotransduction and severe hearing loss. In mice with homozygous mutation of *Tmie* or *Cib2*, MET activity is completely absent due to failures in the assembly or maintenance of the MET apparatus [49,55].

Variants of *PCDH15* and *CDH23* are linked to either ARNSHL, such as DFNB12 and DFNB23 or Usher syndrome type I (USH1) [72,118,[119], [120], [121]]. *PCDH15* is strongly associated with LHFPL5 at the lower end of tip links [28,66]. Mutations in *LHFPL5* are associated with DFNB66/67, a type of severe-to-profound congenital deafness [63,64]. Homozygous mice with *Lhfpl5* mutation show a largely reduction of tip links, likely due to impaired transport or retention of *PCDH15* in stereocilia [28].

Furthermore, several key molecules critical for MET function, such as harmonin, SANS, and MYO7A, are located near CDH23, collectively form the upper tip link density (UTLD) complex. Mutations in these genes can lead to deafness or related syndromes (Table 1). For instance, mutations in harmonin have been associated with Usher syndrome type 1C (USH1C) or DFNB18 [122,123]. Notably, novel heterozygous missense mutations (p.Gly223Cys and p.P234L) in the *USH1C* gene were also identified [124,125], which contribute to ADNSHL, indicating a novel inheritance mode for *USH1C*-related hearing impairment. These mutations impair the interaction between CDH23 and harmonin's PDZ2 domain, disrupting proper assembly and function of the MET channel complex.

Recent research has uncovered additional MET-related gene mutations that lead to deafness. For instance, mutations in *LOXHD1* are responsible for DFNB77 [107,108]. Although MET current in *Loxhd1* mutant inner hair cells initially resemble those of wild-type during the first postnatal week, they experience significant impairment by P11 [105], for reasons that are not yet fully understood. Likewise, mutations in *CLRN1* and *CLRN2* have been linked to Usher syndrome type 3 (USH3) or nonsyndromic recessive deafness [126,127]. Mice lacking *Clrn1* exhibit dysmorphic stereocilia and diminished MET currents, whereas *Clrn2* knockout mice show only reduced MET currents [99,101]. The underlying mechanisms by which these genes influence MET require further investigation.

## 7 Challenges and perspectives

Over the past few decades, the field of hair cell mechanotransduction has made remarkable strides. The identification of several components within the MET machinery, alongside the discovery of TMC1/2 as core elements of the MET channel, has substantially advanced our understanding of the molecular mechanisms driving hair cell mechanotransduction. However, the precise organization of these molecules into a cohesive molecular complex is still being uncovered. Our knowledge of their roles within this protein complex remains incomplete, and there are several critical issues that still need to be addressed. One major challenge is to reconstitute the hair cell MET machinery within a simplified system, such as heterologous cells or an artificial lipid bilayer, which is essential for studying ion channel function directly. Recently, mammalian TMC1 and TMC2 have been successfully expressed in membrane in heterologous cells, and the functional properties of heterologously expressed mammalian TMC1 and TMC2 were characterized. As mammalian TMC1 and TMC2 are mechanotransduction channels, understanding how they are mechanically gated is central for understanding mechanotransduction in hair cells. This would not only deepen our insight into the molecular structure of the MET complex but also offer a valuable tool for more precise investigation of force-driven gating mechanisms. While the cryo-EM structure of the native ion channel complex containing TMC1 has been elucidated in *C. elegans*, the structures are incomplete and preclude a comprehensive understanding of the working mechanism of the MET machinery. We look forward to see the structure of the mammalian channel complex and its interaction with the tip link. Further investigation into the vertebrate MET complex is required to gain a full understanding of the mechanisms of force transduction. Additionally, there remains a lack of clarity regarding how mechanical forces induce conformation changes in the mechanotransduction channel, enabling ion flux via the channel pore. The identification of molecular components within the MET machinery is likely to foster a deeper understanding of these processes. Moving forward, further research is needed to clarify the potential pathological mechanisms of the deafness-related mutations within components of the MET machinery. Over the next decade or two, research in mechanotransduction and hearing is poised for breakthroughs that could significantly advance the field. Advances in technology are rapidly enhancing our understanding of the MET machinery in auditory hair cells. Several emerging techniques, such as single-molecule imaging, cryo-electron tomography (Cryo-ET), and others, hold great potential to overcome the current limitations in MET research. These cutting-edge technologies, individually and in combination, will offer a more comprehensive and dynamic understanding of the MET machinery, allowing researchers to investigate mechanotransduction in greater detail and accuracy. These discoveries are likely to refine our fundamental understanding of auditory transduction while also accelerating the development of new therapeutic approaches for hearing loss.

## Declaration of competing interest

The authors declare that they have no conflicts of interest in this work.
